# *Artificial psychophysics* questions classical hue cancellation experiments

**DOI:** 10.3389/fnins.2023.1208882

**Published:** 2023-07-06

**Authors:** Jorge Vila-Tomás, Pablo Hernández-Cámara, Jesús Malo

**Affiliations:** Image Processing Lab, Parc Científic, Universitat de València, Valencia, Spain

**Keywords:** *artificial psychophysics*, visual neuroscience, hue cancellation experiments, opponent color coding, spectral sensitivity of artificial networks

## Abstract

We show that classical hue cancellation experiments lead to human-like opponent curves even if the task is done by trivial (*identity*) artificial networks. Specifically, human-like opponent spectral sensitivities always emerge in artificial networks as long as (i) the *retina* converts the input radiation into any tristimulus-like representation, and (ii) the post-retinal *network* solves the standard hue cancellation task, e.g. the network looks for the weights of the cancelling lights so that every monochromatic stimulus plus the weighted cancelling lights match a grey reference in the (arbitrary) color representation used by the network. In fact, the specific cancellation lights (and not the network architecture) are key to obtain human-like curves: results show that the classical choice of the lights is the one that leads to the best (more human-like) result, and any other choices lead to progressively different spectral sensitivities. We show this in two ways: through *artificial psychophysics* using a range of networks with different architectures and a range of cancellation lights, and through a *change-of-basis theoretical analogy* of the experiments. This suggests that the opponent curves of the classical experiment are just a by-product of the front-end photoreceptors and of a very specific experimental choice but they do not inform about the downstream color representation. In fact, the architecture of the post-retinal network (signal recombination or internal color space) seems irrelevant for the emergence of the curves in the classical experiment. This result in artificial networks questions the conventional interpretation of the classical result in humans by Jameson and Hurvich.

## 1. Introduction

The classical hue cancellation experiments (Jameson and Hurvich, 1955; Hurvich and Jameson, 1957) are usually considered as the first psychophysical quantification of Hering's intuition on opponent color coding in the human brain (Knoblauch and Shevell, 2004; Stockman and Brainard, 2010; Fairchild, 2013). As an example, an influential textbook on visual neuroscience (Wandell, 1995) introduces hue cancellation as follows: “*Several experimental observations, beginning in the mid-1950s, catapulted opponent-colors theory from a special-purpose model, known only to color specialists, to a central idea in Vision Science. The first was a behavioral experiment that defined a procedure for measuring opponent-colors, the hue cancellation experiment. By providing a method of quantifying the opponent-colors insight, Hurvich and Jameson made the idea accessible to other scientists, opening a major line of inquiry”*.

**Figure 1:**
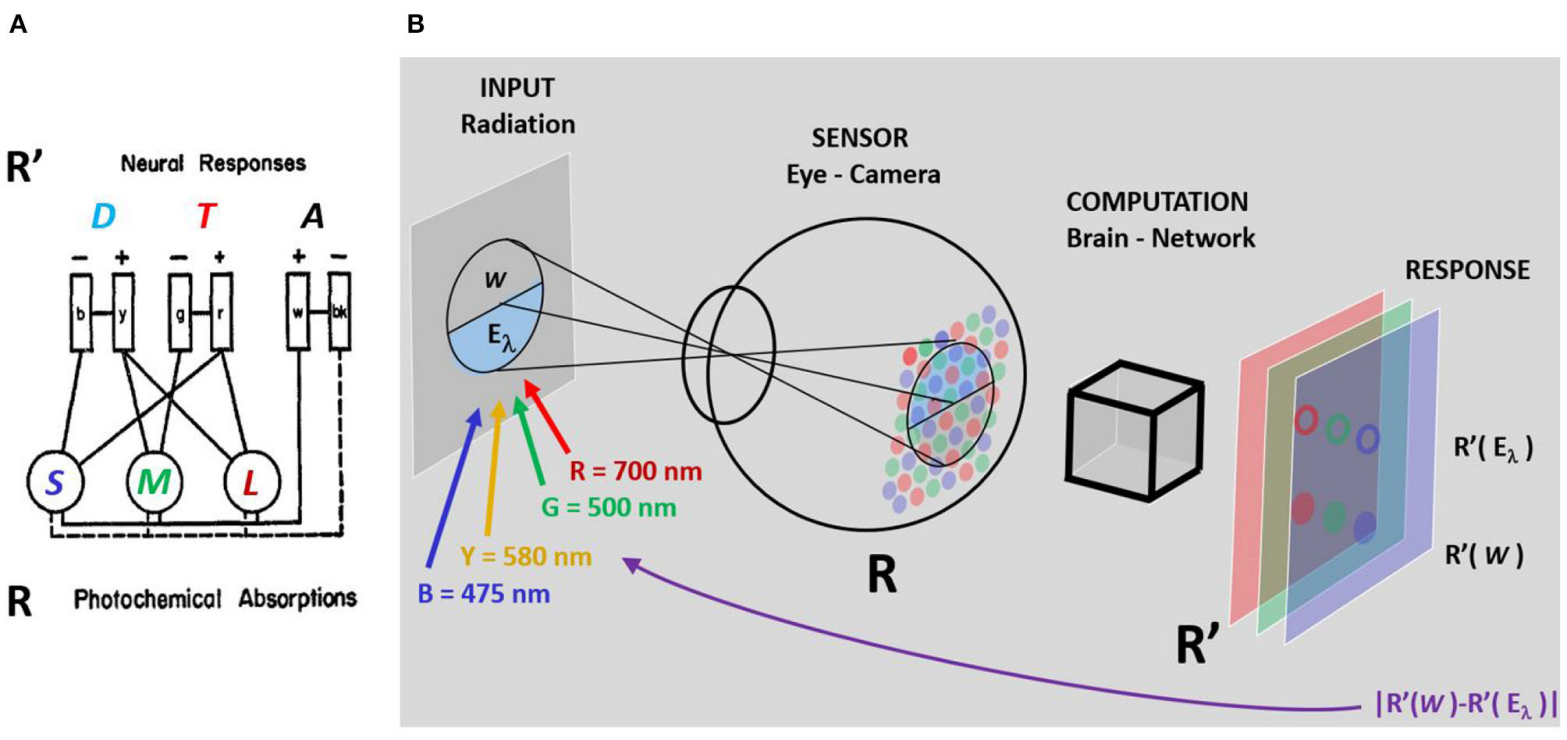
**(A)** Elements of the competing theories of Young-Helmholtz vs. Hering and **(B)** Learning process to get the weights that cancel the hue of certain monochromatic stimulus of wavelength λ. Following the original diagram in Hurvich and Jameson (1957), **(A)** Displays the sensors of the Young-Helmholtz theory, with all-positive sensitivities tuned to *Long, Medium*, and *Short* (LMS) wavelengths, and a possible architecture of a network that would lead to the sensors of the Hering theory: two chromatic sensors with opponent sensitivities, the *Tritanopic* sensor (T) tuned to red-green and the *Deuteranopic* sensor (D) tuned to yellow-blue, together with an Achromatic sensor (A) with a wide all-positive sensitivity. **(B)** Illustrates the hue cancellation experiment: the (natural or artificial) observer *looks for* the weights of the spectral cancelling lights so that a mixture of these cancellation stimuli with the original monochromatic input matches a grey reference (a stimulus with no hue). In this setting, hue cancellation reduces to distance minimization between the responses *R*′ to the white and to the considered λ plus the weighted cancelling lights. The question is whether this search of the weights reveals something about the computation or architecture of the *brain-network* module in **(B)** that transforms *R* into *R*′, or about the nature of the inner color representation *R*′.

The scientific question to be solved by the *hue cancellation experiment* is about the post-retinal neural architecture, or recombination of color signals after photodetection. This is illustrated by Figure 1A, based on the original diagram in Hurvich and Jameson (1957). The authors confront the Young-Helmholtz trichromatic theories of color vision with the qualitative opponent theory of Hering. They propose an architecture to get the Achromatic, Tritanopic (red-green) and Deuteranopic (yellow-blue) sensors (ATD) from the front-end photoreceptors tuned to Long, Medium, and Short (LMS) wavelengths, and hue cancellation would be the tool to quantify the spectral sensitivity of the ATD mechanisms in the proposed architecture.

In this work we present a counter-example based on artificial networks (on automatic differentiation) that suggests that the results of conventional hue cancellation experiments do not provide conclusive information on the inner color representation of the system that mediates the task (the post-retinal network, black box in Figure 1B). Therefore, strictly speaking, the curves from the classical hue cancellation experiments would not be measuring the sensitivity of those ATD mechanisms.

In particular, we show that *identity networks* develop opponent red-green and yellow-blue color valence functions which are quite similar to the human curves independently of the color representation (LMS, RGB or ATD). What we refer to as *identity network* is a trivial architecture whose (3-dimensional) output is exactly the same as its (3-dimensional) input in each spatial location. This trivial network, which already operates in a tristimulus-related representation, [say certain standard LMS cone space (Stockman and Sharpe, 2000), or even in an arbitrary, device dependent, digital count RGB space (Brainard, 1997; Malo and Luque, 2002)] may apply no opponent color coding whatsoever and still gets the human-like curves (in contrast to the specific architecture assumed in Figure 1A). Therefore, the opponent curves that emerge do not strictly inform of the inner (eventually opponent) color representation of the post-retinal neural network. Instead, they are a by-product of the (retinal) tristimulus representation of the input radiation and of the choices in the conventional experimental setting (e.g., the wavelengths of the spectral cancellation lights). To explore in more detail this result, we perform multiple hue cancellation experiments with cancellation lights different to the classical ones and we obtain a clear dependence with the choice of the spectral cancellation lights, achieving the best human-like behaviour only in the case of the classical cancellation lights. This result is confirmed by an analysis of the hue cancellation experiment using a change-of-basis analogy.

## 2. Methods: hue cancellation experiments in artificial networks

### 2.1. General setting

In this work the artificial hue cancellation experiment is a matching problem in the color representation used by the artificial network. Take the setting represented in Figure 1B: for any arbitrary spectral input of wavelength λ, *E*_λ_, and a grey reference, *W*, the network takes the input retinal representation of stimulus and reference, *R*(*E*_λ_) and *R*(*W*), and transforms them into the inner representation $R^{\prime }\left(E_{\lambda }\right)$ and *R*′(*W*). We make no assumption of the nature of this representation *R*′. In Figure 1B *R*′ is represented by red, green and blue layers just for visualization, this does not mean we assume them to be LMS-like. In the initial situation, when no cancelling lights are added, the distance $\left|R^{\prime }\left(W\right)-R^{\prime }\left(E_{\lambda }\right)\right|$ will have a large value. The goal in this matching problem is looking for the optimal weights $w_{\lambda_{c}}^{⋆}\left(\lambda \right)$ of the cancelling lights that minimize the distance between the reference and the monochromatic stimulus plus the weighted cancelling lights:


(1) $$\begin{array}{l} w_{\lambda_{c}}^{⋆}\left(\lambda \right)=\underset{w_{\lambda_{c}}\left(\lambda \right)}{\mathrm{argmin}}|R^{\prime }\left(W\right)-R^{\prime }\left(E_{\lambda }⊕\underset{\lambda_{c}}{\sum }w_{\lambda_{c}}\left(\lambda \right)E_{\lambda_{c}}\right)| \end{array}$$

where the subtraction in the distance is regular subtraction between vectors, but ⊕ stands for additive superposition of radiations. Physical superposition is always positive so, in this case, as conventionally done in color matching experiments (Wyszecki and Stiles, 2000), we assume that *negative* weights in the superposition to *E*_λ_ physically mean the corresponding amount of *positive* superposition to *W*. In short, the cancellation experiment should tell us about the change of color representations, from the input space *R* to the output *R*′. In principle, the goal function in Equation 1 can be applied to regular tristimulus vectors (where vector summation has perceptual meaning) but also to arbitrary, engineering-oriented device-dependent color representations such as digital counts in RGB.

The matching problem described above is just a difference minimization problem which is well suited for learning based on automatic differentiation. In this *artificial psychophysics* setting, the network architecture of the black-box in Figure 1B is fixed but the energy of the cancelling lights (the weights *w*_λ_*c*__) is modified in each iteration to minimize the distance in Equation 1.

Appendix A elaborates on how to approximate monochromatic stimuli for artificial networks intended to work with restricted stimuli such as regular digital images. Appendix B elaborates on how the four individual weighting functions we get from the artificial nets, $w_{\lambda_{c}}^{⋆}\left(\lambda \right)$, are combined into the final valence functions (that happen to be red-green and yellow-blue in the case of the conventional λ_*c*_'s).

### 2.2. Hue cancellation with artificial networks beyond the classical setting

This artificial simulation of the hue cancellation experiment can be applied with any architecture in the fixed network (black box in Figure 1B) and with any choice of λ_*c*_'s for the cancelling lights.

If human-like opponent channels emerge from the simulations even if the network does not have a biologically plausible architecture and independently of the post retinal space, this means that the result of the classical experiment cannot be interpreted as an indication of the existence of post-retinal mechanisms performing the computation suggested in Figure 1A.

Refutation of the conventional interpretation of the classical experiment is stronger if the emergence of opponent curves mainly happens with a particular choice of λ_*c*_'s. This would mean that instead of having the result because of interesting properties of the post-retinal mechanisms, it comes from a fortunate selection of the experimental setting. For this reason it is interesting to simulate hue cancellation for a range of alternative λ_*c*_'s different from the classical experiment.

### 2.3. Differences with the experimental setting for humans

In the original experiments with humans, the cancelling lights had the same energy and their wavelengths were slightly different for the two observers J/H: 467/475 nm (blue), 490/500 nm (green), 588/580 (yellow), and 700/700 nm (red). In all our simulations the cancelling lights always had the same initial energy and we used an equienergetic stimulus as grey reference. In simulating the classical setting, our wavelengths were the ones for observer H (475, 500, 580 and 700 nm). In our experiments we use (without loss of generality) quasi-monochromatic lights so that they can be properly represented in digital values to be processed by conventional artificial networks. These stimuli are defined by a narrow Gaussian spectral radiance added on top of a low-radiance equienergetic background. Appendix A shows examples of these stimuli.

In solving the distance minimization problem, the iterative variation of the weights was applied to the height of the narrow Gaussian of the quasi-monochromatic cancelling lights. These differences (cancelling wavelengths similar to the ones in the classical experiment and narrow-spectrum quasi-monochromatic stimuli) do not imply fundamental differences with the classical setting.

Human observers in the classical experiment do not change all four weights at the same time, but (just for the observers convenience) they just move one at a time (judging how the complementary hue disappears) and repeat the experiment four times. This is not a fundamental difference because (at the expense of longer time per wavelength) after the “first cancellation” the observer could also cancel the remaining hue and then match the response to a grey. Additionally, in any part of the spectrum, is the experimenter in the classical experiment who lets the observer to use “the appropriate” cancellation light. This is not a fundamental difference either because if they could look for the cancellation lights in pairs, simultaneous modification of the opponent cancellation lights would null each other and the effect would be as using a single one.

In the setting that we propose to simulate hue cancellation in artificial systems, the only difference with regard to the experiments in humans is that humans may not need an achromatic reference since they already have the concept of what an achromatic stimulus is, and hence they modify the weights of the cancellation lights to match this mental concept. In the case of artificial systems, obtaining the concept of achromatic reference for hue cancellation is not a problem either. It could be computed from natural images using the classical *grey world assumption* (Finlayson et al., 1998), or simply take a flat spectrum reference as we did here.

### 2.4. The trivial identity network

The counter-example presented in this note is based on a trivial network architecture. Its output is the same as the input: for a color *C*, represented at the input by the array *R*(*C*), the response *R*′(*C*) is just:


(2) $$\begin{array}{l} R^{\prime }\left(R\left(C\right)\right)=I\cdot R\left(C\right)=R\left(C\right) \end{array}$$

This, clearly non-human, trivial architecture preserves whatever previous color representation coming from the sensors. This trivial network is a good counter-example for the eventual human-like results because in the brain, the color representation in the retina certainly changes downstream (Shapley and Hawken, 2011; Shapley, 2019).

## 3. Experiments and results

As stated in the *Methods* section, the conventional interpretation of the classical hue cancellation experiment can be questioned if one finds a counter example showing that human-like opponent valence curves may emerge for the classical choice of λ_*c*_'s regardless of the post-retinal network architecture and color representation. Moreover, refutation would be stronger if one finds that the human-like results are mainly obtained for the classical choice of λ_*c*_'s while other choices lead to progressively different curves regardless of the input color representation space.

According to this, we perform two sets of experiments: (1) we look for counter examples with the classical hue cancellation lights using trivial identity networks working with different color representations (LMS, ATD, and digital RGB). (2) we consider a range of experiments with alternative cancellation lights different from the classical choice using the same trivial identity networks operating either in LMS, ATD, or digital RGB.

### 3.1. Counter examples in the classical setting

In order to check the emergence of human-like curves in hue cancellation even with the trivial identity network, we perform three experiments assuming different *input* representations *R*:

- **Experiment 1:** Identity network working in an arbitrary non-human color representation: a device-dependent digital RGB.
- **Experiment 2:** Identity network working in a standard LMS cone space, as for instance (Stockman and Sharpe, 2000).
- **Experiment 3:** Identity network working in a standard opponent space as for instance, the Jameson and Hurvich model (Jameson and Hurvich, 1955; Capilla et al., 1998).

Note that the above three identity networks would correspond to color representations with quite different qualitative features: (a) if the input is digital RGB, the problem is solved by a system with wide-band overlapping all-positive spectral sensitivities (different from LMS) and compressive nonlinear response in the retina, (b) if the input are standard LMS tristimulus one has a purely linear LMS color code with all-positive sensitivities in the retina, and (c) if the input representation *R* is an opponent system with an achromatic channel and two chromatic channels, the network is fed with a fundamentally different color coding.

**Figure 2:**
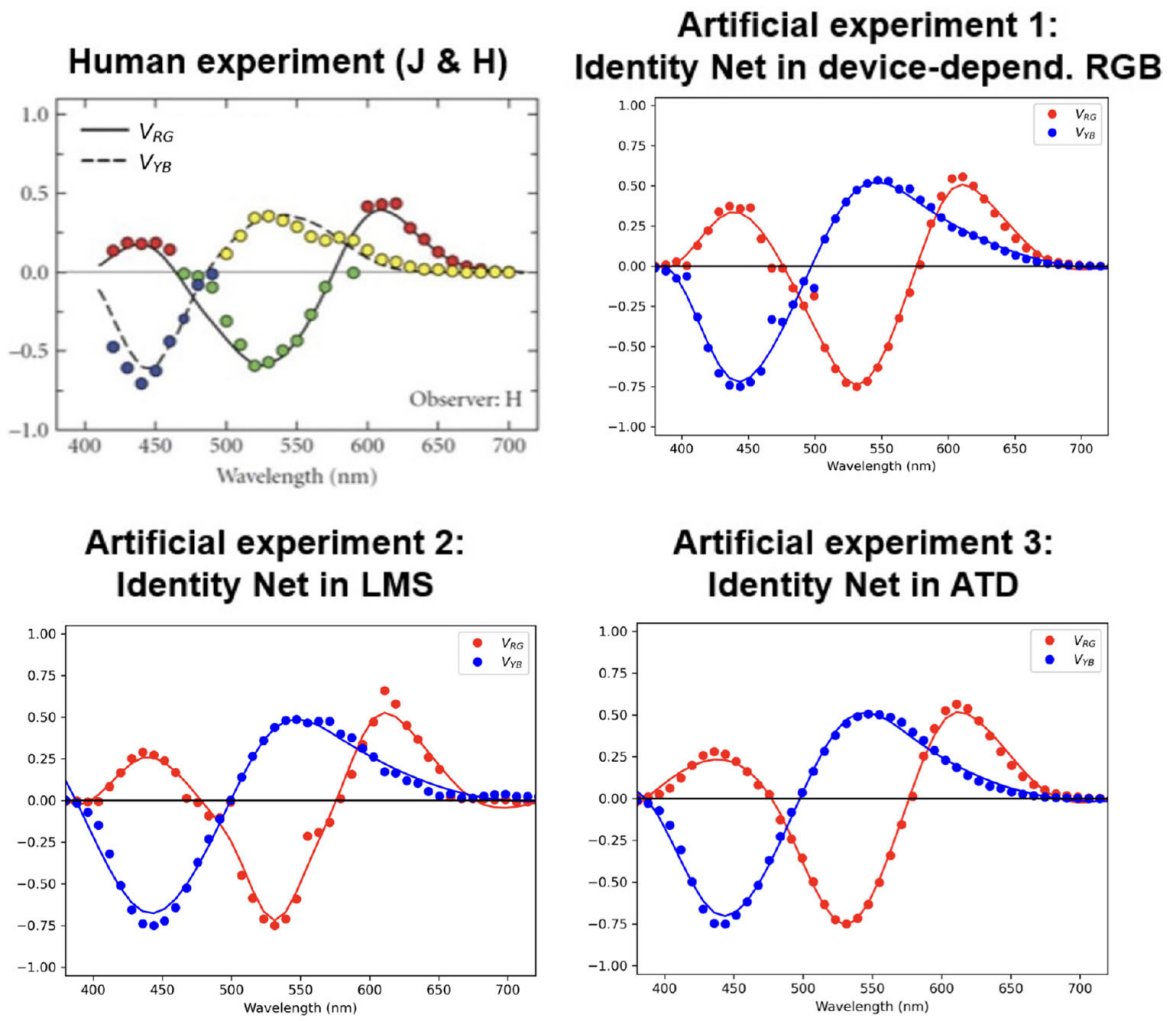
*Main result*: Opponent curves from hue cancellation for the trivial identity network operating in different color representation spaces **(top right and bottom)** compared to the result in humans **(top left)**. The dots represent the result from the artificial matching for each wavelength, and the curves are just smooth fits to the experimental dots.

Figure 2 shows the results of these three hue cancellation experiments together with the experimental results for humans reported in Jameson and Hurvich (1955).

Appendix C shows that (1) the final matches make sense (found at the yellow-blue and red-green curves) and are close to perfect (almost zero difference after the addition of $w_{\lambda_{c}}^{⋆}\left(\lambda \right)E_{\lambda_{c}}$), and (2) the difference minimization process with the different networks is remarkably similar.

The results show that all identity networks, regardless of the space where they operate, lead to similar hue cancellation curves, and these are remarkably similar to the human curves.

### 3.2. Alternative λ_*c*_'s: control experiments and theoretical analysis

The previous artificial experiments question the traditional interpretation of hue cancellation with the classical λ_*c*_'s because not only opponent systems but also trichromatic systems lead to similar opponent results. As anticipated above, the fortunate selection of the cancellation λ_*c*_'s is *somehow* biasing the matching towards the opponent curves.

In order to confirm that this is the case, we propose additional control experiments with artificial networks (experiments 4, 5, and 6), and we introduce a *change-of-basis analogy* of the hue cancellation to understand the results. We show the predictions of this *change-of-basis analogy* in the experiment 7:

- **Experiment 4:** Numerical results of hue cancellation for a range of λ_*c*_'s away from the classical choice using the identity network working in a device-dependent digital RGB space.
- **Experiment 5:** Numerical results of hue cancellation for a range of λ_*c*_'s away from the classical choice using the identity network working in a standard LMS space (Stockman and Sharpe, 2000).
- **Experiment 6:** Numerical results of hue cancellation for a range of λ_*c*_'s away from the classical choice using the identity network working in a standard ATD space (Jameson and Hurvich, 1955; Capilla et al., 1998).
- **Experiment 7:** Exhaustive exploration of (analytical) changes of basis that are similar to hue cancellation experiments for λ_*c*_'s very different from the classical choice.

First, lets introduce the idea of the *change-of-basis analogy* of the hue cancellation experiments, and then we present the results of experiments 4–6 together with the theory-based simulation (experiment 7).

**Figure 3:**
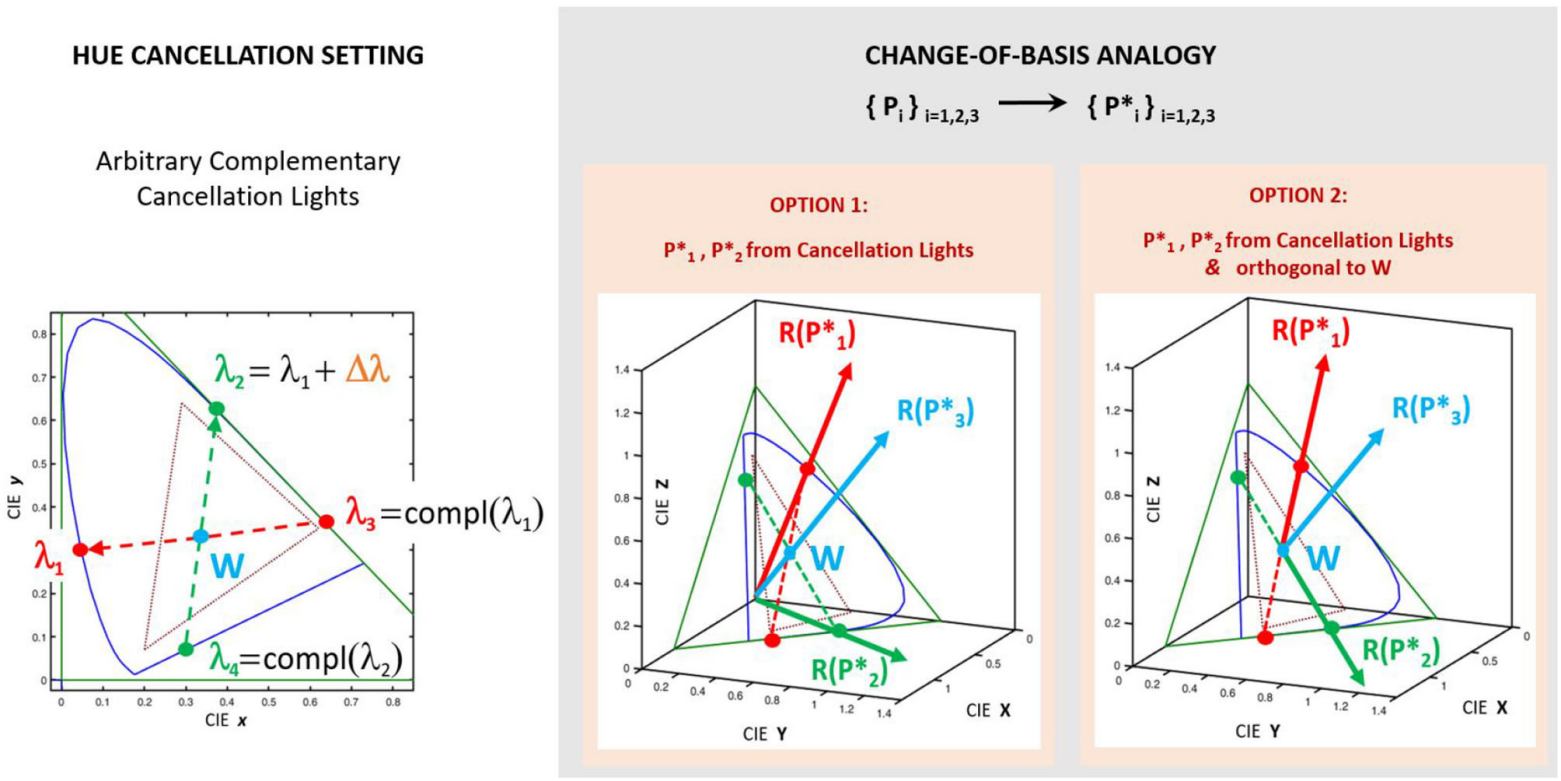
The *change-of-basis analogy:* Hue cancellation experiment as combination of vectors of a new basis. Note that the primaries $P_{i}^{⋆}$ (based on the cancelling lights) are not related to the unknown primaries of the unknown representation *R*′. The primaries $P_{i}^{⋆}$ (either in option 1 or 2) are just an *artifice* to compute analytically the weights $w_{i}^{⋆}\left(\lambda \right)$ from the tristimulus values $R_{1}^{⋆}\left(E_{\lambda }\right)$ and $R_{2}^{⋆}\left(E_{\lambda }\right)$. Given two arbitrary λ_1_ and Δλ, the difference between *option 1* and *option 2* is that in the second the primaries $P_{1}^{⋆}$ and $P_{2}^{⋆}$ are taken to be orthogonal to the one that goes in the direction of the White, $P_{3}^{⋆}\propto W$, so that they convey *less* information about brightness.

Consider the case in which the cancellation lights are complementary in pairs. For instance, in Figure 3, see the pair [λ_1_, λ_3_] and the pair formed by λ_2_ and the magenta referred to as λ_4_. In that situation, the determination of $w_{\lambda_{c}}^{⋆}$ is equivalent to a change to a color basis where two of the primaries go in the directions of the pair of complementary wavelengths (e.g., the red and green vectors in Figure 3). By choosing a third linearly-independent vector (e.g. in the direction of an achromatic color as the vector in blue perpendicular to the triangle of the chromatic diagram) one has a *new basis* of the color space perfectly defined by the *new* primaries, $P_{i}^{⋆}$, with *i* = 1, 2, 3. These *new* primaries are defined by their tristimulus vectors, $R\left(P_{i}^{⋆}\right)$, in the basis of *old* primaries, *P*_*i*_, with *i* = 1, 2, 3. They have chromatic coordinates $r\left(P_{i}^{⋆}\right)$, and, as in every array of chromatic coordinates and tristimulus vectors, they are proportional: $R\left(P_{i}^{⋆}\right)=\gamma_{i}r\left(P_{i}^{⋆}\right)$.

In this situation, taking *P*_*i*_ as the input color representation (as in Figure 1B), hue cancellation with the four lights is analogous to a *change-of-basis* from *P*_*i*_ to $P_{i}^{⋆}$. Therefore, looking for $w_{\lambda_{1}}^{⋆}\left(\lambda \right)$ and $w_{\lambda_{2}}^{⋆}\left(\lambda \right)$ is analogous to the computation of the tristimulus values of the monochromatic components of the equienergetic white $R_{1}^{⋆}\left(E_{\lambda }\right)$ and $R_{2}^{⋆}\left(E_{\lambda }\right)$. Under this *change-of-basis analogy*, the valence functions can be computed analytically from the color matching functions (the vectors *R*(*E*_λ_), ∀λ), and the matrix $M_{PP^{⋆}}$ that changes the vectors from the basis *P*_*i*_ to the basis $P_{i}^{⋆}$:


(3) $$\begin{array}{l} R^{⋆}\left(E_{\lambda }\right)=M_{PP^{⋆}}\cdot R\left(E_{\lambda }\right) \end{array}$$

where, as in any standard change of basis (Wyszecki and Stiles, 2000), the matrix is:


(4) $$\begin{array}{l} M_{PP^{⋆}}={\left(\begin{array}{ccc} R_{1}\left(P_{1}^{⋆}\right) & R_{1}\left(P_{2}^{⋆}\right) & R_{1}\left(P_{3}^{⋆}\right)\\ R_{2}\left(P_{1}^{⋆}\right) & R_{2}\left(P_{2}^{⋆}\right) & R_{2}\left(P_{3}^{⋆}\right)\\ R_{3}\left(P_{1}^{⋆}\right) & R_{3}\left(P_{2}^{⋆}\right) & R_{3}\left(P_{3}^{⋆}\right) \end{array}\right)}^{-1}=\\ \left(\begin{array}{ccc} \gamma_{1}^{-1} & 0 & 0\\ 0 & \gamma_{2}^{-1} & 0\\ 0 & 0 & \gamma_{3}^{-1} \end{array}\right)\cdot {\left(\begin{array}{ccc} r_{1}\left(P_{1}^{⋆}\right) & r_{1}\left(P_{2}^{⋆}\right) & r_{1}\left(P_{3}^{⋆}\right)\\ r_{2}\left(P_{1}^{⋆}\right) & r_{2}\left(P_{2}^{⋆}\right) & r_{2}\left(P_{3}^{⋆}\right)\\ r_{3}\left(P_{1}^{⋆}\right) & r_{3}\left(P_{2}^{⋆}\right) & r_{3}\left(P_{3}^{⋆}\right) \end{array}\right)}^{-1} \end{array}$$

In this *change-of-basis analogy* the hue cancellation valence functions are obtained from the color matching functions in the input representation transformed by the matrix in Equation 3. Note that the weights γ_*i*_ associated to the (arbitrary) length of the vectors, $R\left(P_{i}^{⋆}\right)$, will scale each output $R_{i}^{⋆}\left(E_{\lambda }\right)$. Therefore, despite the shape of the curves is fixed by the matrix of chromatic coordinates of the new basis, the global scale of the predicted functions can be varied via the length of the primaries. As a result, in the simulations using this analogy, given certain cancellation λ_*c*_'s, the length of the basis vectors will be adjusted to obtain the best possible match between the predicted function and the classical curves of Jameson and Hurvich.

As explained in Appendix B, in the settings where the cancelling lights are not strictly complementary (as in the classical setting by Jameson and Hurvich) the curves can be obtained from alternative instrumental lights which are complementary. Then, the contribution of these instrumental lights always can be assigned back to the considered cancelling lights. Therefore, (1) the classical setting can be understood using this *change-of-basis analogy*, and (2) this analogy can be used to explore multiple combinations of axes (λ_1_, λ_3_) and (λ_2_ = λ_1_+Δλ, λ_4_). These configurations can include the original experiment and also other, progressively different, alternatives.

In the experiments 4–6 we execute artificial hue cancellation experiments with identity networks using complementary cancelling lights selected according to the *change-of-basis analogy* described above. We explore a range of λ_1_ over the visible spectrum, and for each λ_1_, we select λ_2_ = λ_1_+Δλ with a range of Δλ so that λ_2_ is still visible. Then, the 3rd and 4th cancellation lights are the complementary lights of λ_1_ and λ_2_. Sometimes the complementary cancellation lights are purple-magenta, as in the arbitrary example of Figure 3, but that is not a conceptual problem to apply the change-of-basis analogy. We take the wavelengths in these control experiments along a uniform grid over the spectral space. The analytical solution of the change-of-basis analogy (Figure 3 and Equation 3) can, of course, be used in this range of λ_*c*_'s. Moreover, its analytical nature implies that one can efficiently sample the spectral space at higher rates. On top of the coarse regular grid shown below, we also perform the artificial hue cancellation at the configurations where the theory predicts better agreement with the opponent curves, which incidentally coincide with the wavelengths chosen in the classic experiment.

**Figure 4:**
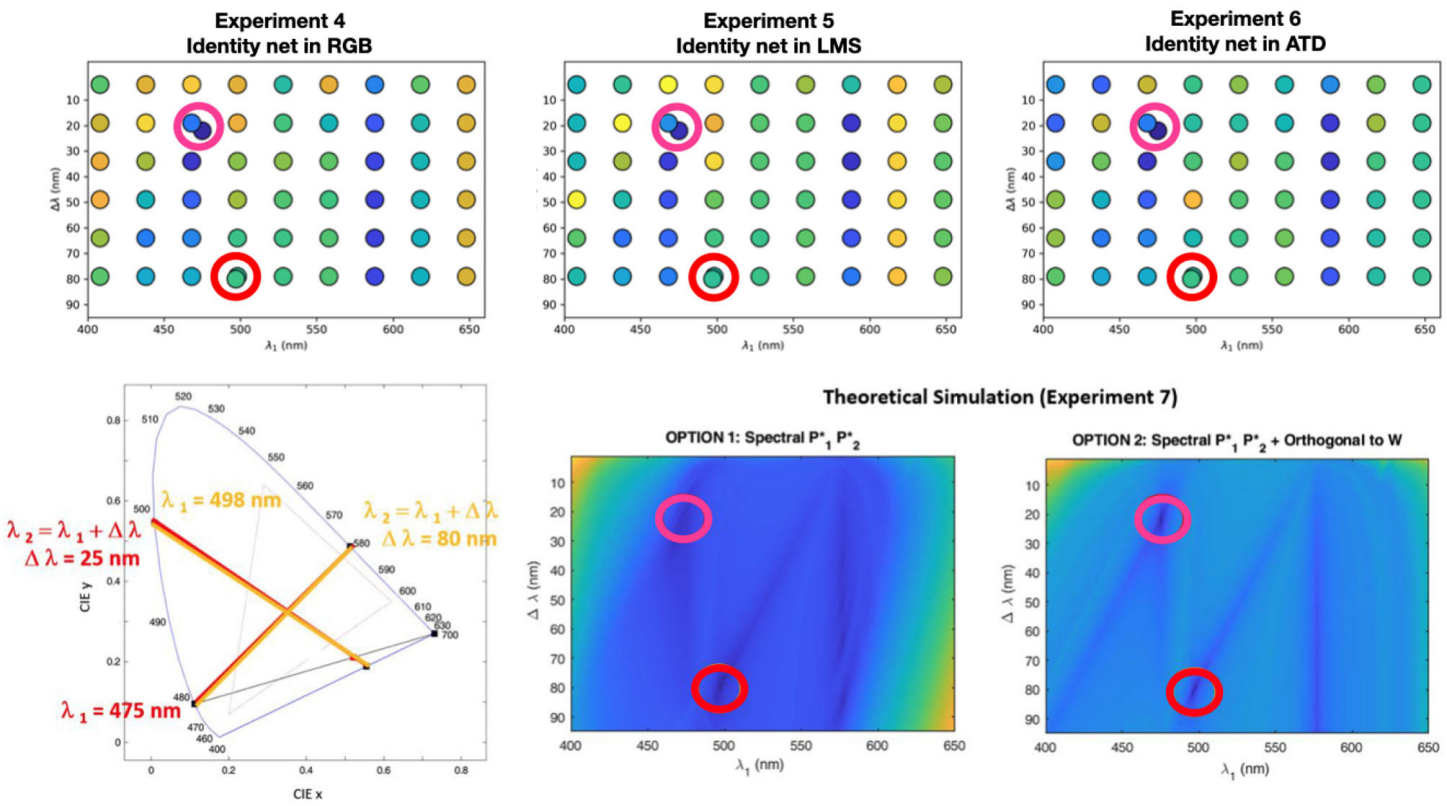
Results of the control experiments (regular grid) together with the results in the original configuration (see the two dots off the regular grid). **Top row** shows the errors of the experiments 4-6 with a blue-yellow colorbar scale where blue means low error (good reproduction of the human opponent curves) and yellow means high departure from the human result. The color code of the departure represents the Mean Squared Error between the human and the artificial curves. **Bottom row (right):** these surfaces represent the same kind of errors, with the same color code for the two options of the change-of-basis analogy. The circles in red and magenta indicate the minima of the theoretical surfaces. **Bottom row (left):** the chromatic diagram shows that the two minima found by the theoretical simulations actually correspond to the same choice of cancellation lights, and coincide with the classical setting (see Appendix B for more information on the auxiliary magenta).

For every considered configuration of cancellation lights we compute the cancellation (or valence) curves and we compute the departure from this result and the human curves of Jameson and Hurvich. Figure 4 shows the error of these predicted valence curves obtained either through the identity networks operating in different color spaces (experiments 4–6), or through the analytical change-of-basis analogy (experiment 7).

The results of experiments 4–7 stress the role of the choice of the cancellation lights in these experiments. Note that *all the error surfaces* have the same specific structure:

- The theoretical surfaces of experiment 7 (which could be densely sampled since they are faster to compute) show two clear minima consistent with the setting selected in the classical experiment. The diagram shows that these two minima are actually equivalent. Moreover, they display a clear pattern of secondary minima. The pattern is more distinct in the setting where the *chromatic* primaries $P_{1}^{⋆}$ and $P_{2}^{⋆}$ are chosen to be orthogonal to the White.
- The errors checked at the grid in the artificial hue cancellation experiments 4-6 are consistent with the theoretical surfaces despite the sampling grid is coarser. The reason for a coarser grid is merely computational^1^. In some cases the deepest minimum is not in the classical point, but the difference is always very small, i.e. in the classical setting the artificial curves are also very similar to the human curves.
- The artificial experiments lead to more marked differences between the agreement in the singular locations of small error (blueish points) and the rest. Note that the errors in the artificial experiments seem to increase faster as one goes away from the regions of small error.

These results (which are consistent regardless of the use of trichomatic representations or opponent representations) suggest that the emergence of the classical curves is more linked to the selection of the cancellation lights than on the inner color representation *R*′.

## 4. Discussion and final remarks

### 4.1. Summary of results

When using trivial (identity) artificial networks in the classical hue cancellation setting, opponent red-green and yellow-blue valence functions emerge regardless of the actual color representation used by the networks (as long as it is a tristimulus representation or even tristimulus-like digital-RGB representations that include mild nonlinearities).

This suggests that these opponent curves do not inform us about the inner workings of the considered system, but about the properties of color mixtures in the tristimulus representations. Given the fact that the mixture of opponent spectral cancellation lights is in the line between them in the chromatic diagram, changing the energy of these cancellation stimuli will always lead to displacements along these lines and hence, proper match with the grey reference (or proper hue cancellation) using the correct proportion of cancellation lights: humans and also trivial machines forced to use spectral (or quasi-spectral) cancellation lights would arrive to the same conclusion.

The reasoning is not as (analytically) obvious in nonlinear representations (as the digital-RGB) but results show that it follows the same trends, thus stressing the generality of the result.

The actual variation of the mixture when modifying the weights in the hue cancellation process only depends on the properties of the additive color mixture, and the path in the diagram is determined by the (classical) choice of the spectral cancellation lights, and not by the inner color representations. Results suggest that a fortunate selection of the cancellation λ_*c*_'s is *somehow* biasing the matching towards the correct opponent curves. If a range of alternative cancellation lights are considered, the results are progressively different from the classical opponent functions.

With the classical λ_*c*_'s, the different color representations only imply different metric spaces to compute the error in the match, but in absence of neural noise (or in moderate neural noise), this would mean minor variations in the result of the minimization, and hence one cannot rule out trichromatic LMS-like representations.

### 4.2. Previous criticisms to hue cancellation experiments

Certainly there are have been a number of well founded criticisms to the classical hue cancellation results. For instance, Wandell (1995) makes this point: to what extent can we generalize from the valence measurements using monochromatic lights to other lights?. If the human behavior for polychromatic light does not follow from the behavior for monochromatic lights, then the data represents only an interesting (but non-generalizable) collection of observations. In general, the linearity assumption is only an approximation (Larimer, 1974; Larimer et al., 1975; Burns et al., 1984; Ayama and Ikeda, 1989; Chichilnisky, 1995). As a result, we need a more complete (nonlinear) model before we can apply the hue cancellation data to predict the opponent-colors appearance of polichromatic lights. Other criticisms refer to overestimation of valence in certain spectral regions in hue cancellation versus other psychophysical methods (Ingling, 1977; Ingling et al., 1978; Ayama and Ikeda, 1989).

However, the problem implied by the systematic emergence of the opponent curves from the identity networks is different. It is not restricted to the linearity assumption. In fact, the systems with nets operating in the LMS or ATD spaces are linear by definition. The emergence of the same result in two different (linear) trivial cases implies that the curves do not give a conclusive message about the inner working of the system.

### 4.3. Consistency with previous results on unique hues

Unique hues are related to the spectral sensitivity of the inner mechanisms that mediate the perception of hue (Webster et al., 2000a; Wuerger et al., 2005). Our results in this work are about the inability to interpret the classical hue cancellation curves as the sensitivity of the inner mechanisms, not about unique hues. However, the invariance that we found in cancellation curves for different internal architectures is consistent with facts observed in previous literature on unique hues.

Studies on unique hues show that they do not depend on the monochromatic or broadband nature of the stimuli used in the measurements: results with monochromatic stimuli (Larimer, 1974; Larimer et al., 1975) coincide with results obtained with display-generated stimuli (Webster et al., 2000a,b; Wuerger et al., 2005) and color chips (Hinks et al., 2007; Shamey et al., 2015). Our results are not in contradiction with that invariance. First, as stated above, our networks used narrowband, but not monochromatic, stimuli. More importantly, the change-of-basis analogy proposed in Section 3.2 and Figure 3 shows that the opponent curves in hue cancellation (minima in the error surfaces in Figure 4) are obtained as long as the chromatic coordinates of the cancellation lights are in the directions of the unique hues. It doesn't matter if they have maximum saturation (monochromatic, as in the analytical computations) or not (broadband, as in the network simulations). Note that the task of the (artificial or human) observer in the experiment is changing the energy (luminance) of the corresponding component and this does not modify the chromatic coordinates of the light (regardless of the saturation or bandwidth of the light).

Moreover, a number of studies (Webster et al., 2000a; Malkoc et al., 2005; Wuerger and Self, 2022) coincide in that there is a weak relation between the unique hues and (small) changes of the sensitivities of the retinal or LGN mechanisms. This is usually interpreted as if hue perception depends on later cortical stages adapted to the environment statistics (Malkoc et al., 2005; Wuerger and Self, 2022). In our case, hue cancellation (optimization of Equation 1) is also independent of the post-retinal recombination. Of course, strong variations on sensitivity (as the reduction of the dimensionality of the color space, as in some dichromats) may have an impact on the cancellation curves, but that is a matter for further research.

We are not saying that the unique hues are a by-product of the cancellation lights. What we say is that the opponent curves from hue cancellation do not inform about the sensitivity of the inner mechanisms, which is a different thing. And this is because once one chooses the cancellation lights (eventually using the information of the unique hues), the result of the cancellation experiment is independent of the neural architecture. Our experiments show that opponent cancellation curves emerge regardless of the sensitivity of the neural mechanisms. Therefore, the opponency in cancellation curves does not tell anything about the actual sensitivity (opponency) of the inner sensors.

Our results are not in contradiction with the invariance of the unique hues using broadband display primaries (Wuerger et al., 2005; Wuerger and Self, 2022) nor broadband Munsell Chips (Hinks et al., 2007; Shamey et al., 2015).

Actually, the change-of-basis analogy proposed in our work (Section 3.2 and Figure 3) shows that the opponent curves in hue cancellation (minima in the error surfaces in Figure 4) are obtained as long as the chromatic coordinates of the cancellation lights are in the directions of the unique hues. It doesn't matter if they have maximum saturation (monochromatic) or not (broadband). Note that the task of the (artificial or human) observer in the experiment is changing the energy (luminance) of the corresponding component and this does not modify the chromatic coordinates of the light (regardless of the saturation or bandwidth of the light).

In fact, independence of our curves on the sensitivity of the retinal and inner mechanisms is consistent with the comments in Webster et al. (2000a), Malkoc et al. (2005), and Wuerger and Self (2022) about the weak relation between unique hues and these sensitivities.

### 4.4. Emergence of human-like opponent curves in artificial systems

Emergence of human-like behavior in artificial systems has been an inspiration for functional (or principled) explanations in theoretical neuroscience (Barlow, 1959, 2001; Dayan and Abbott, 2005). In particular, due in part to the current success of artificial networks in vision tasks (Krizhevsky et al., 2012), there is a growing interest to compare their behavior with humans (Geirhos et al., 2019, 2020; Funke et al., 2021) or with human-like models of traditional visual neuroscience (Martinez et al., 2019; Gomez-Villa et al., 2020; Hepburn et al., 2022; Li et al., 2022; Akbarinia et al., 2023).

In this context, we set a low-level conventional psychophysics program to check the basic behavior of artificial networks in light of known basic human behavior (Hernández-Cámara et al., 2022; Vila-Tomás et al., 2022b). To our surprise, our first experiments with artificial networks (with markedly non-human color representation) actually displayed human-like behavior in hue cancellation (Vila-Tomás et al., 2022a).

That was the origin of this research because the emergence of human-like curves in hue cancellation in networks where opponency had not been built in (nor assumed in the training tasks) could have two implications:

- **Hypothesis A:** On the positive side, it could imply that the considered tasks used to train the nets actually lead to human behavior in scenarios different from the training. These evidences are interesting in the debate about the kind of tasks that may lead to human behavior. Note that certain tasks (e.g., assessing image quality or enhancing the retinal image), may lead to positive or negative results in reproducing human behavior depending on the architecture of the net. Consider examples in Malo and Laparra (2010) and Martinez et al. (2019) for the emergence of contrast nonlinearities, examples in Li et al. (2022) and Akbarinia et al. (2023) for the emergence of the Contrast Sensitivity Functions, or examples in Kumar et al. (2022); Hernández-Cámara et al. (2023) for the visibility of distortions.
- **Hypothesis B:** On the negative side, it could also be that the experimental setting somehow forces the result. In this case the opponent curves would not tell much about the inner color representation of the system, but about the selected *opponent* spectral cancelling lights and about the properties of additive mixtures in tristimulus spaces. These elements (alien to the specific color coding in the network) could also explain the human-like opponent curves.

According to the results reported here, the second hypothesis seems the one that may be true.

### 4.5. Implications in visual neuroscience

Direct physiological recording of the opponent spectral sensitivity of cells (DeValois et al., 1966; Derrington et al., 1984) is (of course) the strongest indication of opponent color coding in the brain. However, following our results with trivial networks, the consistent emergence of the opponent curves in hue cancellation experiments suggests that other psychophysical techniques (Krauskopf et al., 1982) may be more appropriate than hue cancellation to reveal the opponent mechanisms. Definitely, the classical hue cancellation curves cannot be interpreted as a proof of the existence of opponent mechanisms. Similarly, our results suggest that indirect statistical arguments actually give stronger evidences in favour of opponent color coding than hue cancellation. This is consistent with the suggestions on the relevance of adaptation to the statistics of the environment done by Malkoc et al. (2005) and Wuerger and Self (2022) based on the properties of unique hues. Statistical arguments are not limited to classical linear decorrelation (Buchsbaum and Gottschalk, 1983; Ruderman et al., 1998), but also include more recent, nonlinear measures of dependence (MacLeod and von der Twer, 2003; Laparra et al., 2012; Gutmann et al., 2014; Laparra and Malo, 2015).

## Data availability statement

The datasets presented in this study can be found in online repositories. The names of the repository/repositories and accession number(s) can be found below: https://github.com/Jorgvt/PerceptualTests.

## Author contributions

JM set the project of checking the spectral sensitivity of artificial networks and developed the theoretical analogy to obtain analytical results. JV-T envisioned the counter example to refute the classical interpretation of the hue cancellation experiments. PH-C and JV-T built the models and launched the numerical experiments. All authors contributed to the analysis of the results and writing.

## Footnotes

^1^Each location involves the estimation of the two valence curves at 50 λ's. Therefore, it involves 50 hue cancellation experiments, i.e. 50 minimizations, one per λ in the visible range.
