# Supplementary figures and images for: Artificial psychophysics questions classical hue cancellation experiments
Source: Front Neurosci. 2023 Jul 6;17:1208882. doi: 10.3389/fnins.2023.1208882 (PMC10358728; doi:10.3389/fnins.2023.1208882)

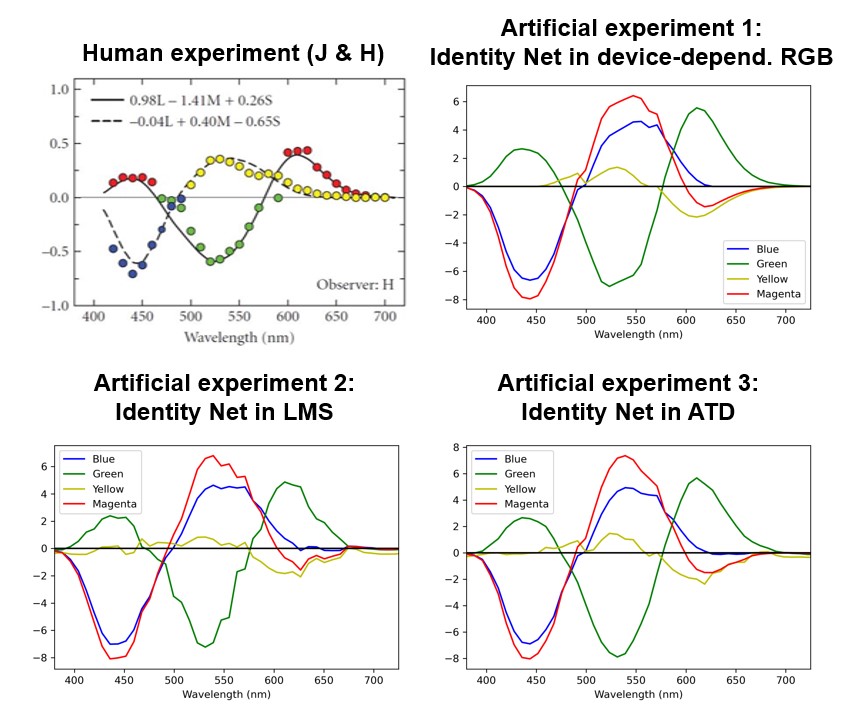

Supplement: Supplementary file 1 [file Data_Sheet_1.zip › 4_curves.jpg]

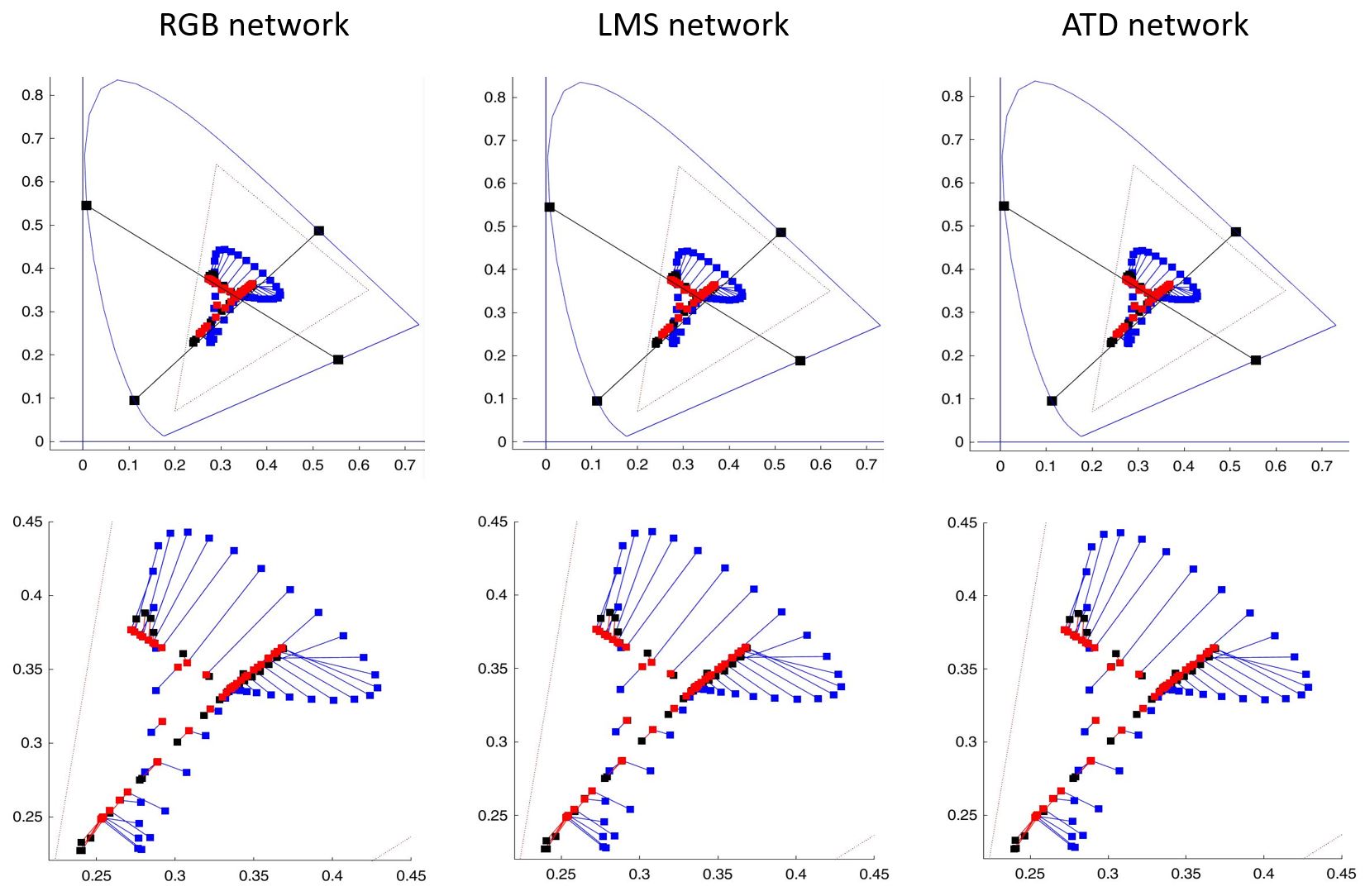

Supplement: Supplementary file 1 [file Data_Sheet_1.zip › matching_results.JPG]

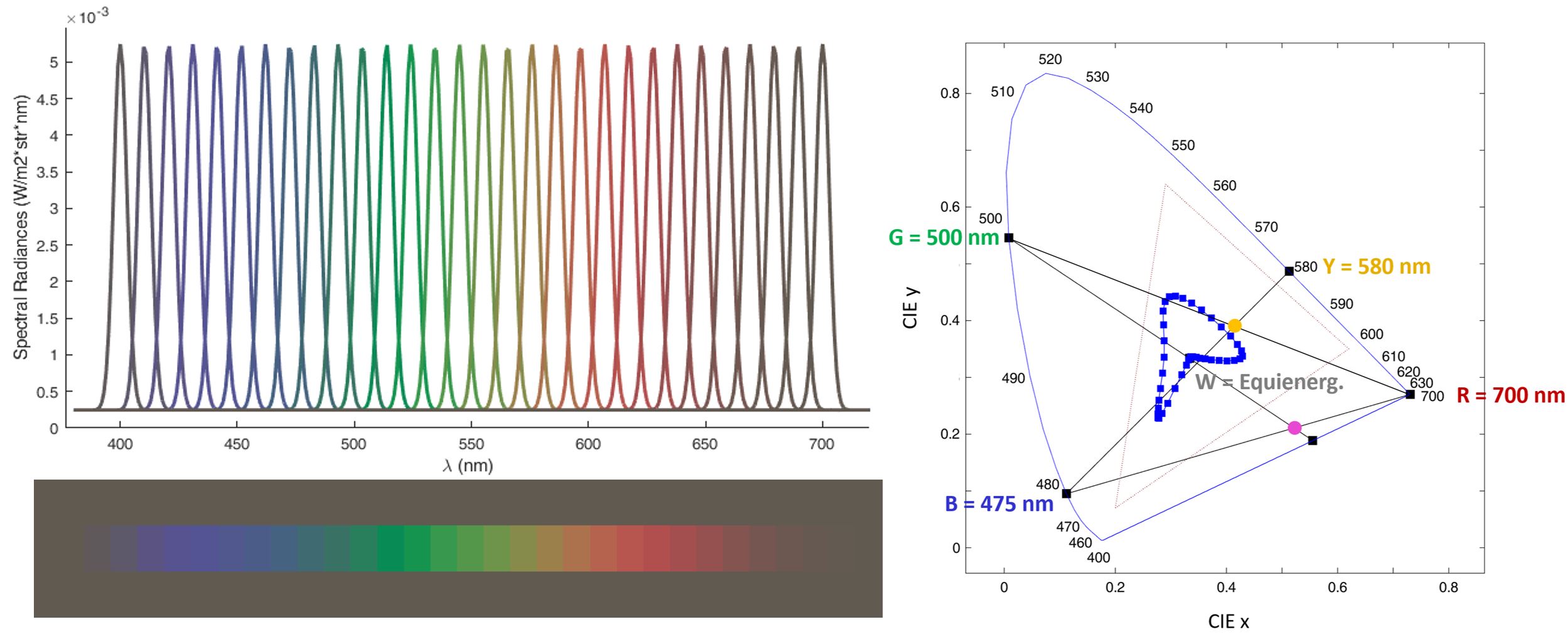

Supplement: Supplementary file 1 [file Data_Sheet_1.zip › appendixAd.JPG]
